# Anti-metabotropic glutamate receptor 5 coexistent anti-N-methyl-D-aspartate receptor encephalitis: a case report and literature review

**DOI:** 10.3389/fimmu.2025.1436246

**Published:** 2025-02-14

**Authors:** Yixin Gu, Tingting Xuan, Pankui Li, Jing Zhou, Zhenhai Wang

**Affiliations:** ^1^ School of Clinical Medicine, Ningxia Medical University, Yinchuan, Ningxia, China; ^2^ Diagnosis and Treatment Engineering Technology Research Center of Nervous System Diseases of Ningxia Hui Autonomous Region, Yinchuan, China; ^3^ Department of Neurology, General Hospital of Ningxia Medical University, Yinchuan, China

**Keywords:** autoimmune encephalitis, metabotropic glutamate receptor 5, antibody overlap, ovarian teratoma, case report

## Abstract

Metabotropic glutamate receptor 5 (mGluR5) antibody encephalitis is an infrequent clinical disorder, initially reported in 2011 among two patients presenting with limbic encephalitis and Hodgkin’s lymphoma. Mental and behavioral abnormalities are prevalent manifestations, accompanied by cognitive impairment, movement disorders, seizures, and other associated symptoms. In this report, we present the case of a young female patient who presented with abnormal mental behavior, seizures, and disturbances of consciousness. A cell-based assay (CBA) showed positive IgG metabotropic glutamate receptor 5 in both her serum and cerebrospinal fluid (CSF), as well as positive IgG N-methyl-D-aspartate receptor (NMDAR) in both her serum and CSF. She was diagnosed with mGluR5 overlapping NMDAR antibody encephalitis and received high-dose intravenous methylprednisolone pulse therapy and immunoglobulin therapy. Tumor screening suggested the presence of bilateral ovarian teratoma. However, unfortunately the prognosis was extremely poor. Clinical results suggested that patients with mGluR5-Abs mostly have good prognoses, excepting our case.

## Introduction

Since the initial identification of anti-N-methyl-D-aspartate receptor (NMDAR) encephalitis in 2007, a plethora of autoantibodies targeting neuronal cell surface or synaptic proteins have been discovered (1). At present, anti-NMDAR encephalitis represents the predominant form, accounting for 54%-80% of AE (2). In 1982, Ian Carr described personality changes and memory loss in his daughter, who was then 15.5 years old. The patient ultimately received a diagnosis of limbic encephalitis and Hodgkin’s lymphoma, which syndrome named “Ophelia syndrome” (3). Metabotropic glutamate receptors (mGluR) are G-protein coupled receptors activated by the binding of glutamate (4). Both mGluR5 and NMDAR are glutamate receptors, which serve as the principal mediators of excitatory synaptic transmission in the brain (5). Metabotropic glutamate receptor 5 (mGluR5) encephalitis is a rare autoimmune encephalitis that was first reported in 2011 in two patients with limbic encephalitis and Hodgkin’s lymphoma (6). Up to now, there have been no reports in China of cases of mGluR5 overlapping NMDAR antibody encephalitis combined with ovarian teratoma. In this report, we present clinical data from a single case of a patient for anti-mGluR5 coexistent anti-NMDAR antibody encephalitis, accompanied by a thorough literature review aiming to enhance clinicians’ understanding of this disease.

## Case presentation

**Figure 1:**
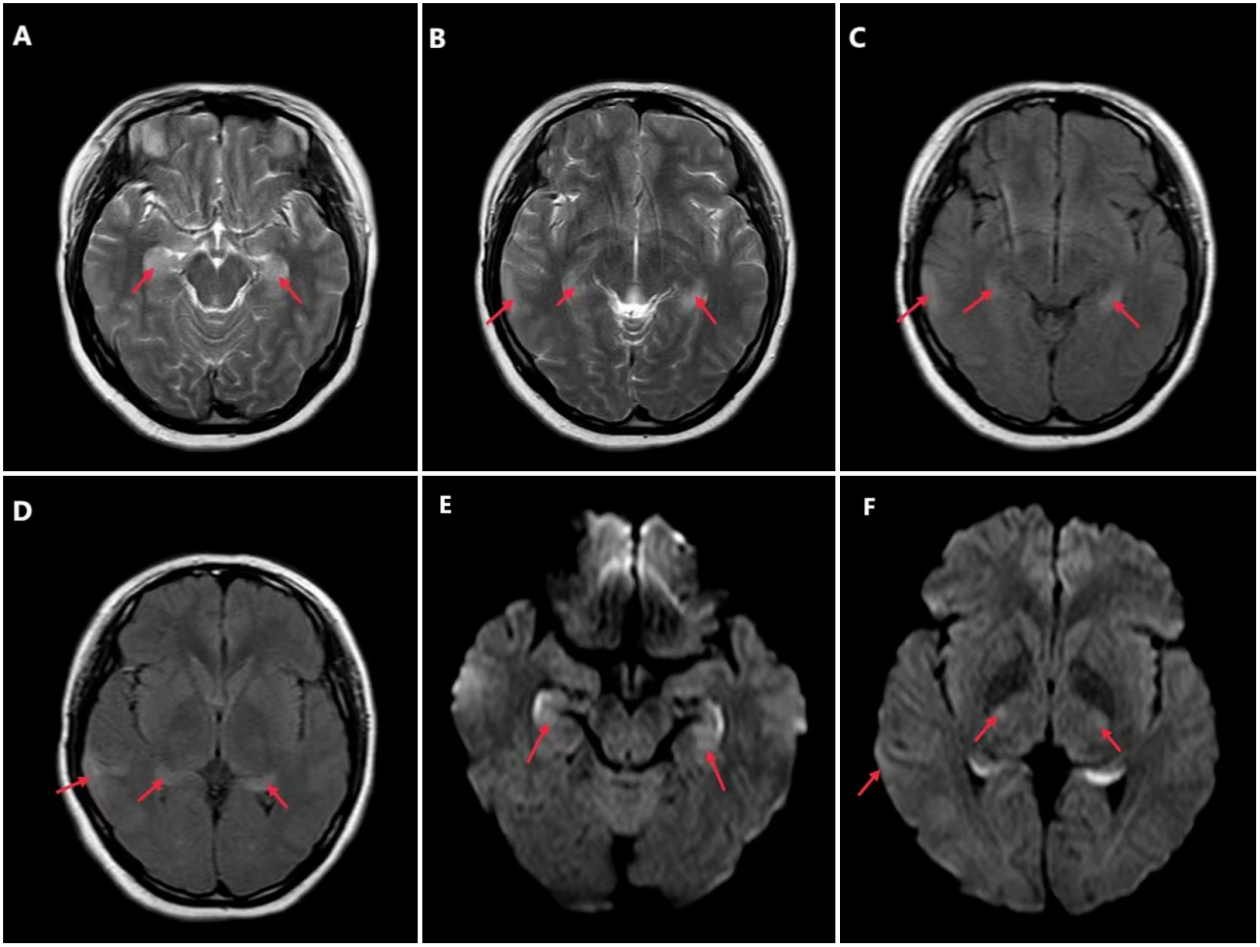
Brain MRI revealed patchy T2WI/FLAIR hyperintensity and abnormally elevated DWI signals in bilateral hippocampus and right temporal parietal lobe (as shown by the arrow). **(A, B)** T2WI, (T2-weighted imaging); **(C, D)** FLAIR, (fluid attenuated inversion recovery); **(E, F)** DWI, (diffusion- weighted imaging).

**Figure 2:**
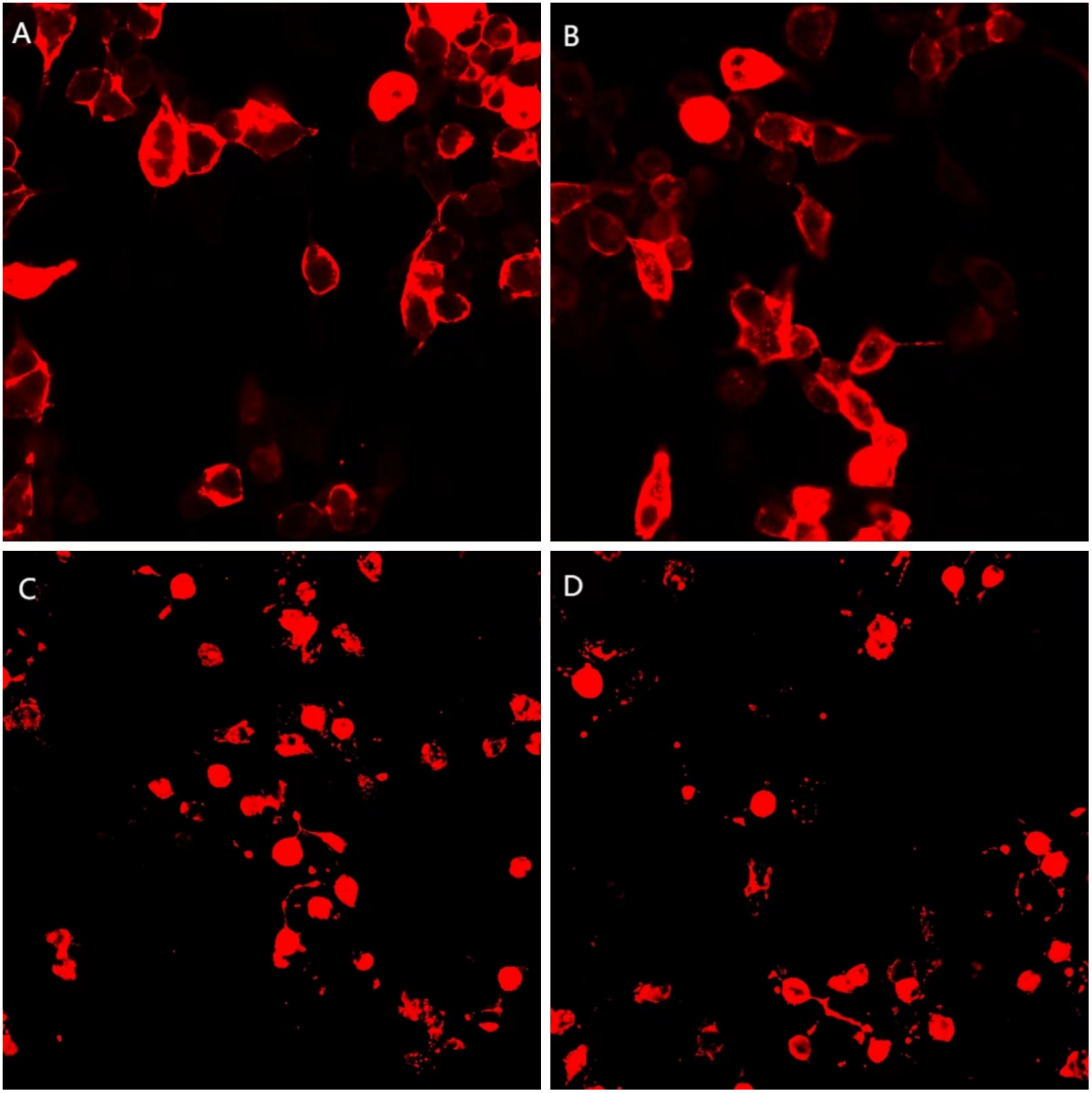
A cell-based assay (CBA) demonstrates positive mGluR5 (1:30, 1:30) and NMDAR (1:100, 1:100) expression in both serum and CSF. **(A, C)** serum; **(B, D)** CSF; **(A, B)** mGluR5 antibody; **(C, D)** NMDAR antibody.

A 21-year-old female patient was first admitted to the hospital on June 16, 2022, with “mental and behavioral abnormalities for a duration of 5 days, and convulsions in all limbs for 2 days.” The patient has no prior medical or familial history of neuropsychiatric disorders. On June 11, 2022, the patient exhibited neuropsychiatric symptoms characterized by disorganized speech, tangentiality, and agitation. On June 14, 2022, limb convulsions accompanied by pyrexia were observed. Subsequently, on June 15, 2022, there was an alteration in consciousness, which should not be referred to. The probability of considering AE is significantly augmented subsequent to admission. Therefore, on June 18, 2022, she received high-dose intravenous methylprednisolone pulse therapy (1000 mg/d for 3 days, 500 mg/d for 3 days, 80 mg/d for 3 days), and subsequent administration of epilepsy control medications (intramuscular phenobarbital sodium injection, sodium valproate, levetiracetam tablets, etc.), along with endotracheal intubation and mechanical ventilation. Unfortunately, the patient’s family members request discharge, and the patient was subsequently discharged on June 27, 2022. The patient was readmitted on July 13, 2022, presenting with the aforementioned symptoms. In the cerebrospinal fluid, leukocytes were 140/mm^3^, 94% of lymphocytes, the red blood cell count was 100/mm^3^, the protein concentration was 0.36 g/L, and the glucose concentration was 3.2 mmol/L. However, the outcome of Pandy test is positive. Brain magnetic resonance imaging (MRI) revealed patchy T2WI/FLAIR hyperintensity and abnormally elevated DWI signals in bilateral hippocampus and right temporal parietal lobe (**Figure 1**). Lumbar puncture was performed again, and the repeated CSF test still indicated leukocytosis (127/mm^3^), a low protein level (0.11 g/L), and a high glucose concentration (8.4 mmol/L). A cell-based assay (CBA) showed seropositive antibodies of NMDAR (1:100) and mGluR5 (1:30), as well as in the CSF for both NMDAR (1:100) and mGluR5 (1:30) (**Figure 2**). 24-hours ambulatory electroencephalogram (EEG): multiple sharp slow waves and spike rhythms activity in the right frontal region. The pelvic enhanced CT scan showed that mixed density shadow in bilateral adnexal area and the bottom of the uterus, with an increased size measuring approximately 3.8cm x 4.6cm. Additionally, there was an elevated CA199 level of 50.60U/ml. Unfortunately, the family declined to undergo biopsy and surgical intervention. One month later, the pelvic enhanced CT revealed an increase in size of the lesions compared to previous findings: bilateral adnexal areas exhibited mixed low-density shadows, suggestive of teratoma (right side measuring 3.6 cm x 3.0 cm, left side measuring 4.9 cm x 5.4 cm).

**Figure 3:**
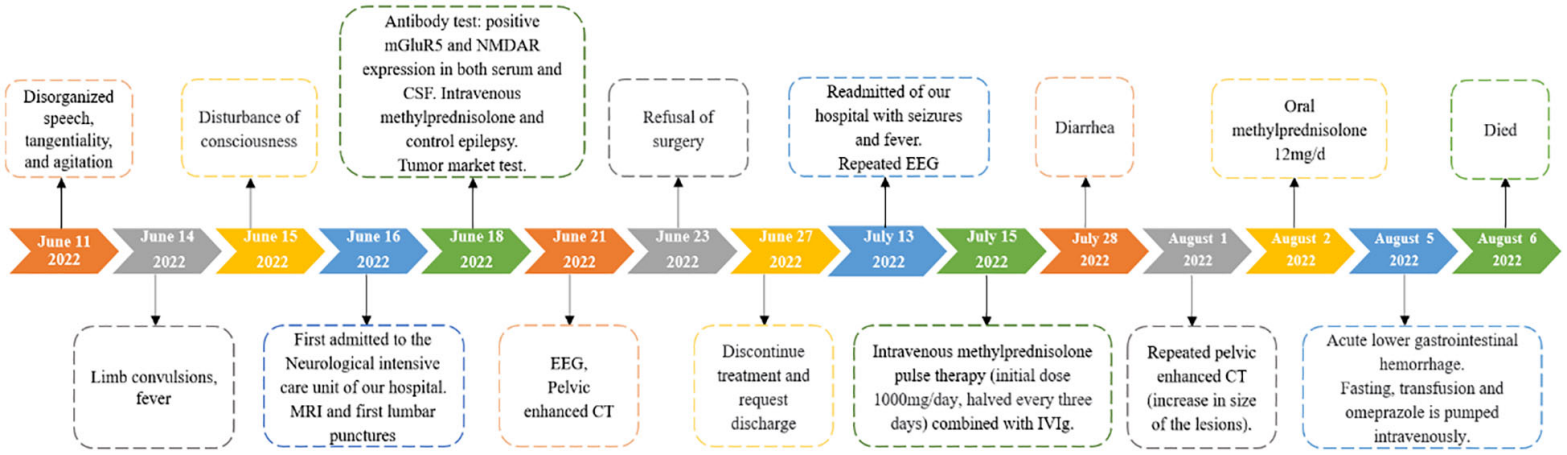
Timeline with clinical manifestations, diagnosis and therapeutic interventions.

Based on the patient’s medical history, presenting symptoms, and positive findings of mGluR5 and NMDAR autoantibodies, she has finally been diagnosed with mGluR5 overlap NMDAR antibody-associated AE. Therefore, she received high-dose intravenous methylprednisolone pulse therapy (1000 mg/d for 3 days, 500 mg/d for 3 days, 120mg/d for 3 days, 60mg/d for 3 days, 40mg/d for 3 days, 20mg/d for 3 days), Following, she is prescribed oral prednisolone (12mg/d) for maintenance therapy. The patient’s condition remains critical, and human immunoglobulin (25g/d for 5 days) is being used in combination for immune enhancement. However, the patient’s condition was complicated. Throughout this period, the patient consistently experienced high fever, with a temperature peaking at 40°C, and underwent continuous renal replacement therapy (CRRT) treatment. Subsequently, acute heart failure developed in the patient along with an elevated NT-proBNP level of 14700pg/ml. Consecutive sputum, blood, and ascites cultures demonstrated the presence of Acinetobacter baumannii, Candida albicans, and Pseudomonas aeruginosa strains successively. Based on drug sensitivity testing results and pharmacist recommendations, appropriate antibiotic therapy was administered. Unfortunately, the patient succumbed to acute gastrointestinal hemorrhage during the course of hospitalization. The clinical manifestations, diagnosis and therapeutic interventions of the patient have been summarized in **Figure 3**.

## mGluR5 antibody CBA

mGluR5-IgG in serum was tested by a cell-based assay (CBA). HEK293 cells were co-transfected with full-length human mGluR5 and pcDNA3.1-EGFP. After 36 hours of transfection in 96 well plate, the cells were fixed with 4% paraformaldehyde for 20 minutes, washed with phosphate-buffered saline (PBS) and permeabilized with 0.1% Triton X-100 in PBS for 20 minutes, which was ready for antibody detection. Serum diluted at 1:10 in PBS-10% goat serum and incubated on cells for 2 hours at room temperature. Cells were then washed in PBS-0.1% Tween 20 for 3 times, incubated for 30 minutes with goat anti-human IgG (1:500, Thermo Scientific), washed again in PBS-0.1% Tween 20, and evaluated by immunofluorescence microscopy. Two independent masked assessors classified each sample as positive or negative based on the intensity of immunofluorescence in direct comparison with non-transfected cells and control samples. Once confirmed, the positive specimens were then serially diluted by threefold from 1:100 to 1:1000 to determine the titers. The final titer was defined as the sample dilution value for which specific fluorescence was barely but clearly identifiable and expressed as the corresponding dilution value.

mGluR5-IgG in CSF was tested by a cell-based assay using HEK293 cells co-transfected with human mGluR5 and pcDNA3.1-EGFP. Thirty-six hours after transfection the HEK293T cells were fixed with 4% paraformaldehyde for 20 minutes and permeabilized with 0.1% Triton X-100 in phosphate-buffered saline (PBS) for 20 minutes. Cells were incubated with patient’s CSF for 2 hours and then immunolabeled with an AlexaFluor 546 secondary antibody against human IgG (1:1000, Thermo Scientific) for 1 hour at room temperature. Images were acquired using a Zeiss Axiovert A1 fluorescence microscope.

## Discussion

In this study, we present a case of AE characterized by the coexistence of double antibodies targeting mGluR5 and NMDAR in conjunction with bilateral ovarian teratoma. The occurrence of Anti-mGluR5 encephalitis is infrequent in clinical practice, and there is a scarcity of reports on the coexistence of mGluR5 with other antibody-associated encephalitis. To date, a total of six cases of mGluR5 overlapping with other antibody encephalitis (**Table 1**) have been reported (7–11), with four cases demonstrating co-occurrence of NMDAR antibodies (8, 10, 11); among these, one case was positive for MOG, NMDAR, and mGluR5 antibodies (8), while another case showed positivity for NMDAR, AMPAR, and mGluR5 antibodies (10). Additionally, one case tested positive for LGI1 antibody (9), and another case exhibited positivity for SOX1 antibody (7). The injection of IgG from patients with anti-mGluR5 encephalitis into the ventricular system of mice has been demonstrated in animal studies to induce memory impairment and heightened anxiety, while concurrently diminishing mGluR5 levels within the hippocampus (12). Notably, mGluR5 plays a pivotal role in memory acquisition by primarily augmenting NMDAR response (13). Both mGluR5 and NMDAR are glutamate receptors, which serve as the principal mediators of excitatory synaptic transmission in the brain (5, 14). Glutamate receptors (GluRs) can be categorized into ionic (iGluRs) and metabolic receptors (mGluRs) (15). Anti-NMDAR encephalitis is a clinical syndrome characterized by the presence of patients with anti-mGluR5 encephalitis into the ventricular system of mice has been demonstrated in animal studies to induce memory impairment and heightened anxiety, while concurrently diminishing mGluR5 levels within the hippocampus (12). Notably, mGluR5 plays a pivotal role in memory acquisition by primarily augmenting NMDAR response (13). Both mGluR5 and NMDAR are glutamate receptors, which serve as the principal mediators of excitatory synaptic transmission in the brain (5, 14). Glutamate receptors (GluRs) can be categorized into ionic (iGluRs) and metabolic receptors (mGluRs) (15). Anti-NMDAR encephalitis is a clinical syndrome characterized by the presence of iGluRs antibodies (16). Previous studies have indicated that mGluR5 encephalitis commonly coexists with NMDAR encephalitis, suggesting potential shared pathogenic mechanisms or possibly due to the relatively high incidence of anti-NMDAR encephalitis. The rare syndrome of marginal encephalitis associated with iGluRs antibody also encompasses the presence of an uncommon anti-AMPAR antibody (17). The primary clinical presentations observed in patients with mGluR5 overlapping NMDAR antibody encephalitis in this study encompassed cognitive and behavioral abnormalities, seizures, and impaired consciousness, exhibiting no significant deviations from previous studies. It has been reported in the literature that the patient with overlapping LGI1 antibodies showed left faciobrachial dystonic seizures, which represent the primary clinical manifestation of LGI1 antibody encephalitis. The patient ‘s mGluR5 serum titer was 1:10, while CSF analysis negative results, thus LGI1 was considered to be a pathogenic antibody. Patients with overlapping MOG antibodies showed cerebral cortical encephalitis, which was easily misdiagnosed as viral encephalitis. Patients with overlapping SOX1 antibodies have progressive ophthalmoplegia and cerebellar ataxia symptoms, alongside small cell lung cancer. Anti-SOX1 antibodies are regarded as serum biomarkers for small cell lung cancer (18). The overlapping antibody encephalitis of NMDAR, AMPAR and mGluR5 showed refractory epilepsy.

**Table 1:** Clinical characteristics, tumor status, treatment and prognosis of 6 patients with mGluR5 antibody overlapping encephalitis.

No	Age (y)	Sex	Prodromal symptoms	Cardinal symptom	CSF	Brain MRI	EEG	mGluR5 antibody	Other antibodies	Tumor	Tumor marker	Treatment	Prognosis
1	21	F	–	Acute behavioral abnormalities accompanied by consciousness disorders, limb convulsions, and fever	140WBC/mm³, IgG↑	Bilateral hippocampus and right temporal parietal lobe T2/FLAIR high signal.	Multiple sharp slow waves and spike rhythms activity in the right frontal region.	CSF: 1:30 S: 1:30	NMDAR antibody CSF: 1:10 S: 1:100	Bilateral ovarian teratoma	CA199: 50.6U/ml↑	IV methylprednisolone, IVIg, Antiepileptic, antiviral, blood transfusion, hemodialysis, anti-infection, mechanical ventilation	Death after 56 days of hospitalization treatment.
2	38	M	Headache, fever	Difficulty in language expression. Seizures, mental disorders	396WBC/mm³, protein↑	Wide cortical DWI/FLAIR in the left hemisphere of the brain high signal.	Diffuse sharp slow wave activity in the left frontal lobe and temporal lobe.	CSF: 1:10 S: 1:10	MOG antibody CSF: 1:10 S: 1:32 NMDAR antibody CSF:1:10 S: (-)	-	-	IV methylprednisolone, IVIg, antiepileptic, antiviral, antipsychotic, oral prednisolone, oral mycophenolate mofetil	Follow-up for 6 Months, complete recovery. MOG antibody (S: 1:10)
3	35	F	Headache	Acute spatial disorientation memory deficits, visual hallucinations, seizures, unconsciousness dystonia	120WBC/ mm^3^, IgG↑	Bilateral hippocampus T2/FLAIR high signal.	Focal slow wave, epileptic discharge.	CSF: 1:100 S: 1:10	NMDAR antibody (CSF+S) AMPAR1- AMPAR2 (CSF)	Mature teratoma	–	IV methylprednisolone, IVIg, oral prednisolone, surgery	Follow-up for 6 months, death.
4	26	F	–	Acute episodes of mental and behavioral abnormalities accompanied by irritability, mania, gibberish sleepwalking, hallucinations	4WBC/mm^3^, protein↑	The splenium of corpus callosum showed low signal on T1WI. T2/FLAIR/DWI high signal.	Diffuse slow wave.	CSF: (-) S: 1:10	NMDAR antibody CSF: 1:3.2 S: 1:32	Bilateral ovarian teratoma	AFP:51.6 ng/ml↑ CA125:266. 8U/ml↑	IV methylprednisolone, IVIg, oral prednisolone, surgery, chemotherapy	Follow-up for 3 Months, complete recovery. NMDAR antibody (S: 1:10)
5	65	F	–	Sudden left facial-brachial dystonia episodes and no response	Normal	Right caudate nucleus and putamen have low signal on T1WI, and T2/FLAIR high signal.	Diffuse slow wave.	CSF: (-) S: 1:10	LGI1 antibody CSF: 1:30 S: 1:100	–	–	IV methylprednisolone, IVIg, antiepileptic, oral prednisolone	Follow-up for 3 months, complete recovery.
6	75	M	Weight loss	Progressive ophthalmoplegia, hypertonia, executive dysfunction, positional tremor, gait instability	6WBC/ mm^3^, IgG↑	Bilateral middle temporal lobe T2/FLAIR high signal.	-	CSF: 1:320 S: 1:160	SOX1 antibody	Small cell lung cancer	–	IV methylprednisolone, IVIg, chemotherapy radiotherapy	Follow-up for 62 Months, cognitive improvement, no changes in ophthalmic muscle paralysis.

F, female; M, male; IgG, Immunoglobulin G; MRI, Magnetic Resonance Imaging, DWI, diffusion- weighted imaging; T1WI, T1-weighted imaging; T2/FLAIR, fluid attenuated inversion recovery; EEG, electroencephalogram; S,serum; CSF, Cerebrospinal Fluid; mGluR5, metabotropic glutamate receptor 5; NMDAR, N-methyl-D-aspartate receptor; MOG, myelin oligodendrocyte glycoprotein; LGI1, Leucine-rich Gliom-nactivated-1; AMPAR, α-Amino-3-Hydroxyl-5-Methyl-4-Isoxazolepropionic acid receptor; SOX1, Sex-determining region Y box protein 1; AFP, alpha fetoprotein; IV, intravenous; IVIg, intravenous immunoglobulin.

Anti-NMDAR encephalitis was first discovered in 2007 (3), which is the most common autoimmune encephalitis. Its primary clinical manifestations encompass mental and behavioral abnormalities, cognitive impairment, seizures, autonomic dysfunction, and disturbances in consciousness (19). The symptoms of anti-NMDAR encephalitis exhibit a wide range of manifestations. Some patients initially present with a solitary neurological or psychiatric symptom, while additional symptoms may progressively emerge over the course of several months. The occurrence of anti-mGluR5 antibody encephalitis was initially documented in 2011, when it was observed in two patients with limbic encephalitis and Hodgkin’s lymphoma. However, mGluR5 antibody encephalitis remains a rare condition in clinical practice. As the detection of antibodies becomes more widespread and our understanding of the disease improves, there has been a gradual increase in case reports. The association between mGluR5 antibody encephalitis and Hodgkin’s lymphoma has been reported in study (6, 19). However, our findings suggest that while this condition is indeed associated with tumors, there is no evidence of a specific link to Hodgkin’s lymphoma. This discrepancy may be attributed to the presence of overlapping antibodies or variations in genetic backgrounds. Previous research has indicated that anti-mGluR5 encephalitis be accompanied by peripheral neuropathy (20), suggesting that mGluRs antibodies can impact not only the central nervous system but also the peripheral nervous system (PNS). It is widely acknowledged that NMDAR antibody encephalitis exhibits a strong association with ovarian teratomas and demonstrates a higher prevalence among young female patients. A systematic review of studies conducted between 2007 and 2020 concluded that the incidence of ovarian teratoma among patients diagnosed with anti-NMDAR encephalitis is 37.4% (21). In this case, the clinical presentations of epileptic seizures and psychiatric abnormalities may be attributed to the presence of both mGluR5 and NMDAR antibodies; however, it is challenging to definitively determine which antibody exerts a predominant influence in this patient.

AE is a rare neurological disorder, and its diagnosis is on the rise due to the growing prevalence of autoantibody testing and enhanced awareness among clinicians. Nevertheless, it is undeniable that the differential diagnosis of AE occurs more frequently than the condition itself, encompassing a range of disorders such as central nervous system (CNS) infections, metabolic encephalopathy, primary central nervous system lymphoma, Creutzfeldt-Jakob disease (CJD), mitochondrial diseases (22).

Patients diagnosed with AE frequently exhibit prodromal symptoms, including headache and fever (notably in cases of anti-NMDAR encephalitis), which can complicate the differential diagnosis from infectious etiologies. Among infectious etiologies, viruses represent the most significant pathogens identified, with herpes simplex virus encephalitis being the predominant form of viral encephalitis. Kong (22) have developed and validated a novel diagnostic model that effectively differentiates viral encephalitis (VE) from autoimmune limbic encephalitis (ALE). Compared to patients whose conditions are driven by immunological factors, patients with infectious etiologies are more predisposed to experience headache, nausea/vomiting, and elevated white blood cell counts in the cerebrospinal fluid, while exhibiting a lower likelihood of status epilepticus. Furthermore, individuals diagnosed with viral encephalitis may present at an older age and demonstrate fewer neuropsychiatric symptoms than their counterparts with autoimmune encephalitis (23). Specific diagnosis is achieved through the detection of herpes simplex virus pathogens in both serum and CSF. MRI of the brain can be useful in the differential diagnosis of encephalitis. Most patients with AE have either bilateral or unilateral increased T2/FLAIR signals in the medial temporal lobes without contrast enhancement or abnormal DWI (24).

Temporal lobe gliomas that fail to exhibit a sustained response to immunotherapy, demonstrate a mass effect on MRI, and lack an inflammatory response in CSF can help differentiate them from autoimmune encephalitis (25). Primary central nervous system lymphoma (PCNSL) exhibits characteristics similar to AE, including unilateral involvement of the medial temporal lobe on MRI, elevated lymphocyte counts in CSF, and heightened sensitivity to steroid therapy. A definitive diagnosis necessitates histopathological examination (26). Mitochondrial encephalomyopathy with lactic acidosis and stroke-like episodes (MELAS) can initially manifest with altered consciousness and psychiatric symptoms, elevated cerebrospinal fluid cell counts, and focal lesions in the lateral temporal lobe on MRI. Negative CSF neuronal surface antibodies, MRS findings, and muscle biopsy results will necessitate a more detailed differential diagnosis (27).

Similar to other forms of antibody-associated AE, most patients with anti-mGluR5 encephalitis showed good responses to the immunotherapy (10). Anti-mGluR5 encephalitis should be treated with first-line immunotherapies, which include high-dose intravenous corticosteroids, intravenous immunoglobulin, and plasmapheresis (28). For severe cases, a combination of corticosteroids shock and intravenous immunoglobulin therapy may be employed (29). For patients who do not respond adequately to first-line immunotherapy, the consideration of second-line immunotherapy using agents such as rituximab, cyclophosphamide, or mycophenolate mofetil is warranted (30). Meanwhile, symptomatic and supportive treatments were administered based on clinical manifestations, encompassing the management of psychotic symptoms and antiepileptic therapy. Previous research has demonstrated that upon detection of an ovarian teratoma, prompt surgical intervention is imperative. Delaying the excision of a teratoma beyond one month is correlated with adverse clinical outcomes (31).

Previous research has indicated that the majority of patients with mGluR5 antibody encephalitis exhibit a favorable prognosis upon follow-up, with only a minority experiencing an unfavorable outcome (32). Compared to patients with good outcomes, patients with bad outcomes had higher frequency of hypoventilation and higher severity at peak of the disease, reflected by higher mRS score (10). The results of a large observational study on the prognosis of anti-NMDAR encephalitis indicate that (33) within the initial 24 months, 78.6% of the patients had a good prognosis, 12% relapsed, and 5.9% died. Titulaer et al. (33) demonstrated that factors associated with a favorable prognosis included non-admission to the ICU, early initiation of treatment, and lower disease severity within the first four weeks of onset. Schubert et al. (34) highlighted that the incidence of autonomic dysfunction and the utilization of mechanical ventilation during hospitalization were significantly correlated with adverse neurological outcomes at discharge, and these factors may potentially contribute to unfavorable long-term prognosis (35).

Unfortunately, the patient in this study exhibited a poor prognosis. Previous literature has indicated that surgical intervention can significantly enhance the prognosis. Due to concerns regarding the potential impact on the patient’s fertility, following initial hospitalization, the family declined surgical intervention and discontinued immunotherapy, subsequently choosing to leave the hospital of their own accord. Upon readmission, the optimal window for surgical treatment had elapsed, and the patient’s condition was critically severe. The primary cause of mortality for the patient was hemorrhagic shock caused by lower gastrointestinal hemorrhage. The pathogeny of lower gastrointestinal hemorrhage was evaluated as follows: Severe infection results in bone marrow suppression and abnormal coagulation function, with the patient’s coagulation function repeatedly reported as critical value during hospitalization. The patient had multiple systemic infections, including hematogenous infection, pulmonary infection, urinary tract infection, and abdominal infection, may experience intestinal flora imbalance due to the use of various antibiotics. Moreover, the patient has intermittent diarrhea, it is not excluded that antibiotic-related diarrhea leads to complications such as intestinal wall edema, inflammation, bleeding and even necrosis. Hemorrhage resulting from rupture of intestinal vascular malformation: during the course of the disease, the patient suddenly had a large amount of bloody stool, with a significant amount of bleeding and the possibility of intestinal vascular malformation was not excluded. Administration of high-dose hormone pulse therapy may also lead to gastrointestinal hemorrhage. The patient was admitted to the hospital in a critical condition, presenting numerous complications including septic shock and potential stress-induced gastrointestinal mucosal bleeding.

This study is a single case report and has certain limitations in assessing the efficacy of immunotherapy and prognosis. To date, only case reports of mGluR5 antibody-overlapping patients with encephalitis have been documented, and the lack of large-scale studies has resulted in an inadequate evaluation of long-term outcomes and the effect of immunotherapy.

## Conclusions

We reported a case of fatal mGluR5 overlapping NMDAR antibody-associated AE with bilateral ovarian teratoma, which widens the understanding of this disease. This case underscores the hazards of severe AE and emphasizes the need for clinicians to be vigilant in identifying severe encephalitis, particularly in instances where mGluR5 coexists with other encephalitis.

## Data Availability

The raw data supporting the conclusions of this article will be made available by the authors, without undue reservation.
