# Supplementary material for: Anti-metabotropic glutamate receptor 5 coexistent anti-N-methyl-D-aspartate receptor encephalitis: a case report and literature review
Source: Front Immunol. 2025 Feb 14;16:1436246. doi: 10.3389/fimmu.2025.1436246 (PMC11867952; doi:10.3389/fimmu.2025.1436246)
Supplement: Supplementary file 1 [file DataSheet1.pdf]

This report form has been electronically signed and authenticated.

金域医学 KingMed Diagnostics

Kingmed Diagnostics Ningxia
Results of Test Report

Specimen barcode	6400475485	Hospital	Ningxia MedicalUniversity General Hospital	Experiment number	ZM15S0028
sex	female			Sample	CSF
Age	21 years old	Department	NCU	Sample collection time	June 16, 2022

Project	Test Method	Result	Reference value
anti-IgLON5 antibody IgG	CBA	(－)	(－)
anti-DPPX antibody IgG	CBA	(－)	(－)
anti-mGluR1 antibody IgG	CBA	(－)	(－)
anti-NMDAR antibody IgG	CBA	(＋) 1:100	(－)
anti-AMPAR1 antibody IgG	CBA	(－)	(－)
anti-AMPAR2 antibody IgG	CBA	(－)	(－)
anti-LG1 antibody IgG	CBA	(－)	(－)
anti-CASPR2 antibody IgG	CBA	(－)	(－)
anti-GABAB antibody IgG	CBA	(－)	(－)
anti-GABAA antibody	CBA	(－)	(－)
anti-mGluR5 antibody IgG	CBA	(＋) 1:30	(－)
anti-GAD65 antibody IgG	CBA	(－)	(－)
anti-MOG antibody	CBA	(－)	(－)
anti-GFAP antibody	CBA	(－)	(－)
anti-AQP4 antibody	CBA	(－)	(－)

Application and Interpretation:

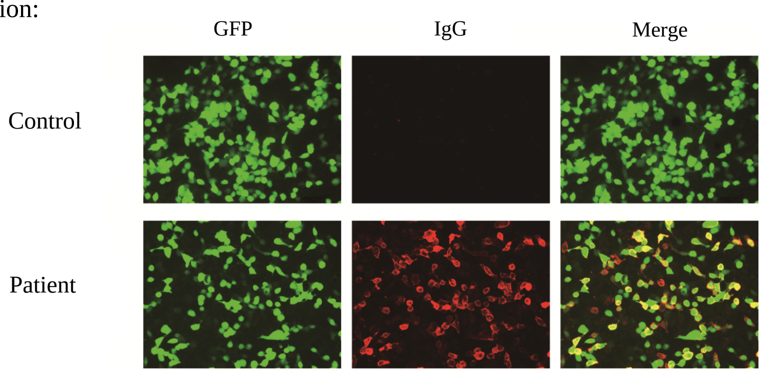

Figure A：Examples of negative and positive controls for antibody detection using the double fluorescence cell transfection (CBA) method.

Testing principle: The cell-based assay (CBA) for indirect immunofluorescence involves transfecting the autoimmune encephalitis antigen gene into mammalian cells, leading to specific expression of the corresponding antigen and co-expression of green fluorescent protein (GFP) as an internal reference. Subsequently, the antigen-expressing transfected cells are fixed on 96-well plates to form antigen arrays, enabling semi-quantitative detection of specific antibodies in human serum, plasma, or cerebrospinal fluid samples based on the principle of indirect immunofluorescence.

Result judgment: Under fluorescence microscope observation, first use the green light channel to observe the transfection situation of the cells. If the plasmid has been successfully transfected, you can observe that the cells have green fluorescence (such as the GFP channel in the figure). Then use the red light channel to observe. If you observe that the cell membrane of the transfected cells in the sample well has a relatively obvious red fluorescence (such as the positive control of IgG in the figure), it is a positive sample for the antibody; if you observe that the cell membrane of the transfected cells in the sample well has no relatively obvious red fluorescence or that untransfected cells have red fluorescence, etc., it is a negative sample (such as the negative control of IgG in the figure). The result can be further confirmed by overlapping the green channel and red channel.

Main Check 侯莉 Reviewer 梁惠玉 Authorize 曾嘉风 Primary Testing Laboratory: Minte Biology Test report Date: June 18, 2022

Website: www.kingmed.com.cn

GZ002MNSUN5HQVK

This report form has been electronically signed and authenticated.

Kingmed Diagnostics Ningxia
Results of Test Report

Specimen barcode	6400475485	Hospital	Ningxia MedicalUniversity General Hospital	Experiment number	ZM15S0028
sex	female			Sample	CSF
Age	21years old	Department	NCU	Sample collection time	June 16, 2022

B

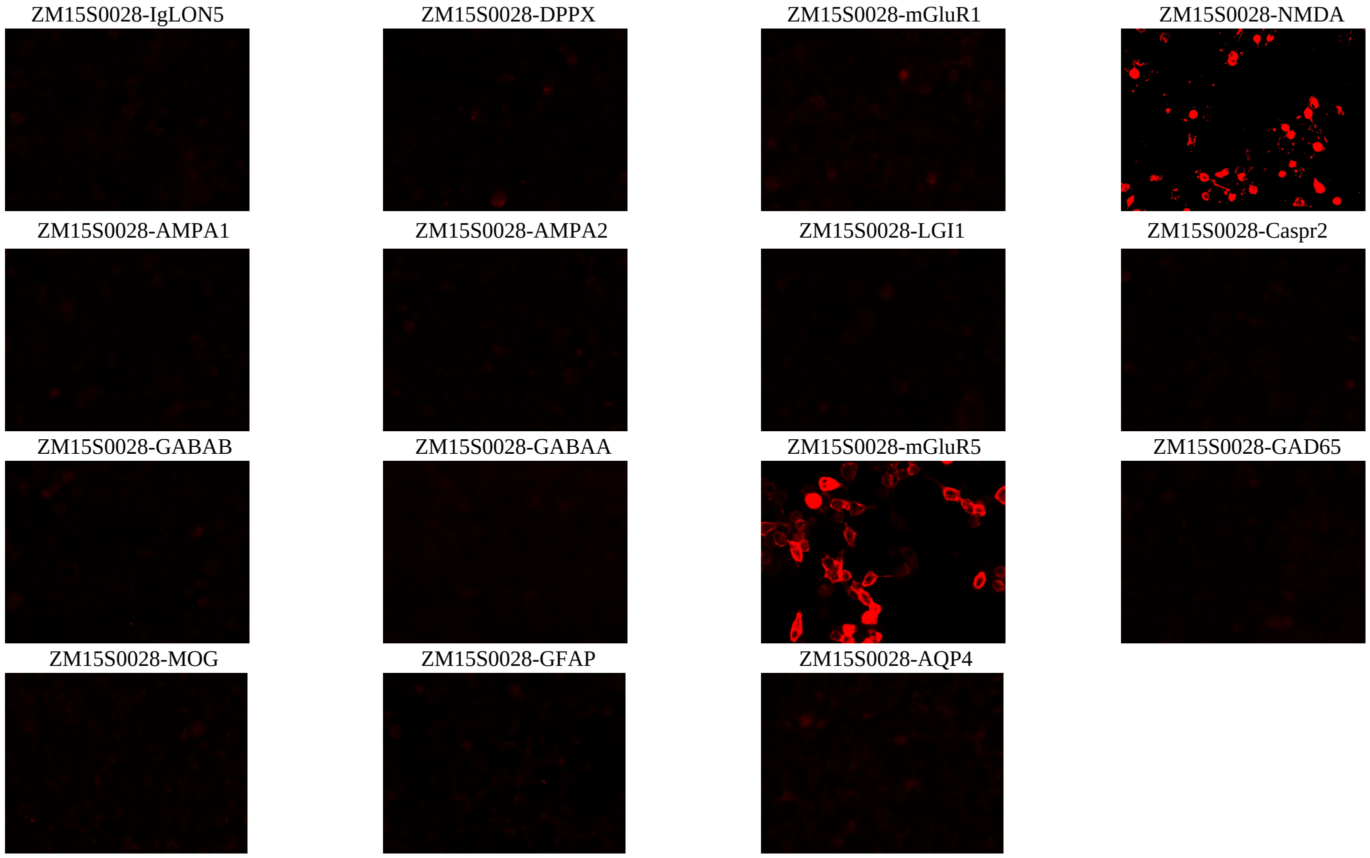

Figure B： The results of the fluorescence map of the sample.

Test Results: The results of the red channel image are displayed, and the negative or positive results are first determined according to the above judgment criteria. If the sample is a positive result, 3-5 fields are selected under the microscope and compared with the red fluorescence of the control sample to give the positive titer value by comparing the intensity of the red fluorescence with that of the control sample.

CBA IIFT Result interpretation (serum)		
Antibody titers results	The 1:10 dilution ratio can be observed to have a fluorescence intensity.	Compare the fluorescence intensity with the positive standard of 1:100 and 1:320 titer.
negative	Negative	--------
1:10	Pianissimo	Fluorescence intensity far＜1:100
1:30	Piano	Fluorescence intensity＜1:10
1:100	Strong	Fluorescence intensity=1:100
1:300	Fortissimo	Fluorescence intensity=1:300
1:1000	Fortissimo	Fluorescence intensity＞1:300

CBAIIFT Result interpretation (CSF)		
Antibody titers results	The un-diluted condition can be observed in the fluorescence intensity.	Compare the fluorescence intensity with the positive standard of 1:100 and 1:320 titer.
negative	Negative	--------
1:1	Pianissimo	Fluorescence intensity far＜1:100
1:10	Piano	Fluorescence intensity＜1:100
1:30	Moderate	Fluorescence intensity＜1:100
1:100	Strong	Fluorescence intensity=1:100
1:300	Fortissimo	Fluorescence intensity=1:300
1:1000	Fortissimo	Fluorescence intensity＞1:300

Main Check 侯莉 Reviewer 梁思玉 Authorize 曾喜凤 Primary Testing Laboratory: Minte Biology

Test report Date: June 18, 2022

Website: www.kingmed.com.cn

GZ002MNSUN5HQVK

This report form has been electronically signed and authenticated.

Kingmed Diagnostics Ningxia
Results of Test Report

Specimen barcode	6400475486	Hospital	Ningxia MedicalUniversity General Hospital	Experiment number	ZM15S0029
sex	female			Sample	serum
Age	21 years old	Department	NCU	Sample collection time	June 16, 2022

Project	Test Method	Result	Reference value
anti-IgLON5 antibody IgG	CBA	(−)	(−)
anti-DPPX antibody IgG	CBA	(−)	(−)
anti-mGluR1 antibody IgG	CBA	(−)	(−)
anti-NMDAR antibody IgG	CBA	(+) 1:100	(−)
anti-AMPAR1 antibody IgG	CBA	(−)	(−)
anti-AMPAR2 antibody IgG	CBA	(−)	(−)
anti-LG1 antibody IgG	CBA	(−)	(−)
anti-CASPR2 antibody IgG	CBA	(−)	(−)
anti-GABAB antibody IgG	CBA	(−)	(−)
anti-GABAA antibody	CBA	(−)	(−)
anti-mGluR5 antibody IgG	CBA	(+) 1:30	(−)
anti-GAD65 antibody IgG	CBA	(−)	(−)
anti-MOG antibody	CBA	(−)	(−)
anti-GFAP antibody	CBA	(−)	(−)
anti-AQP4 antibody	CBA	(−)	(−)

Application and Interpretation:

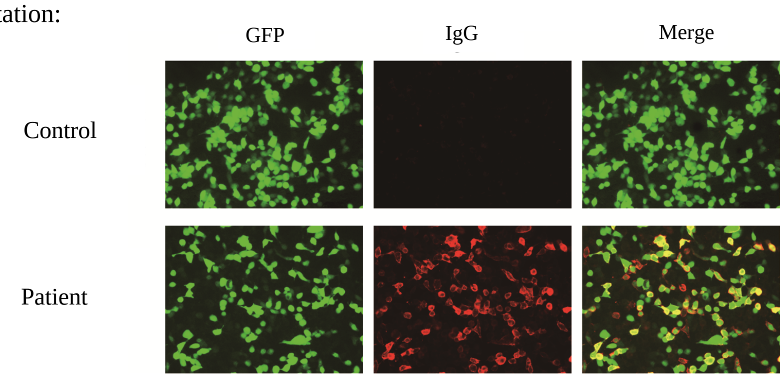

Figure A：Examples of negative and positive controls for antibody detection using the double fluorescence cell transfection (CBA) method.

Testing principle: The cell-based assay (CBA) for indirect immunofluorescence involves transfecting the autoimmune encephalitis antigen gene into mammalian cells, l eading to specific expression of the corresponding antigen and co-expression of green fluorescent protein (GFP) as an internal reference. Subsequently, the antigen-exp ressing transfected cells are fixed on 96-well plates to form antigen arrays, enabling semi-quantitative detection of specific antibodies in human serum, plasma, or cerebrospinal fluid samples based on the principle of indirect immunofluorescence.

Result judgment: Under fluorescence microscope observation, first use the green light channel to observe the transfection situation of the cells. If the plasmid has been successfully transfected, you can observe that the cells have green fluorescence (such as the GFP channel in the figure). Then use the red light channel to observe. If you observe that the cell membrane of the transfected cells in the sample well has a relatively obvious red fluorescence (such as the positive control of IgG in the figure), it is a positive sample for the antibody; if you observe that the cell membrane of the transfected cells in the sample well has no relatively obvious red fluorescence or that untransfected cells have red fluorescence, etc., it is a negative sample (such as the negative control of IgG in the figure).The result can be further confirmed by overlapping the green channel and red channel.

Main Check 侯莉 Reviewer Authorize Primary Testing Laboratory:Minte Biology Test report Date: June 18, 2022

Website: www.kingmed.com.cn

GZ002MNSU3KYEDJ

This report form has been electronically signed and authenticated.

Kingmed Diagnostics Ningxia
Results of Test Report

Specimen barcode	6400475486	Hospital	Ningxia MedicalUniversity General Hospital	Experiment number	ZM15S0029
sex	female			Sample	serum
Age	21years old	Department	NCU	Sample collection time	June 16, 2022

B

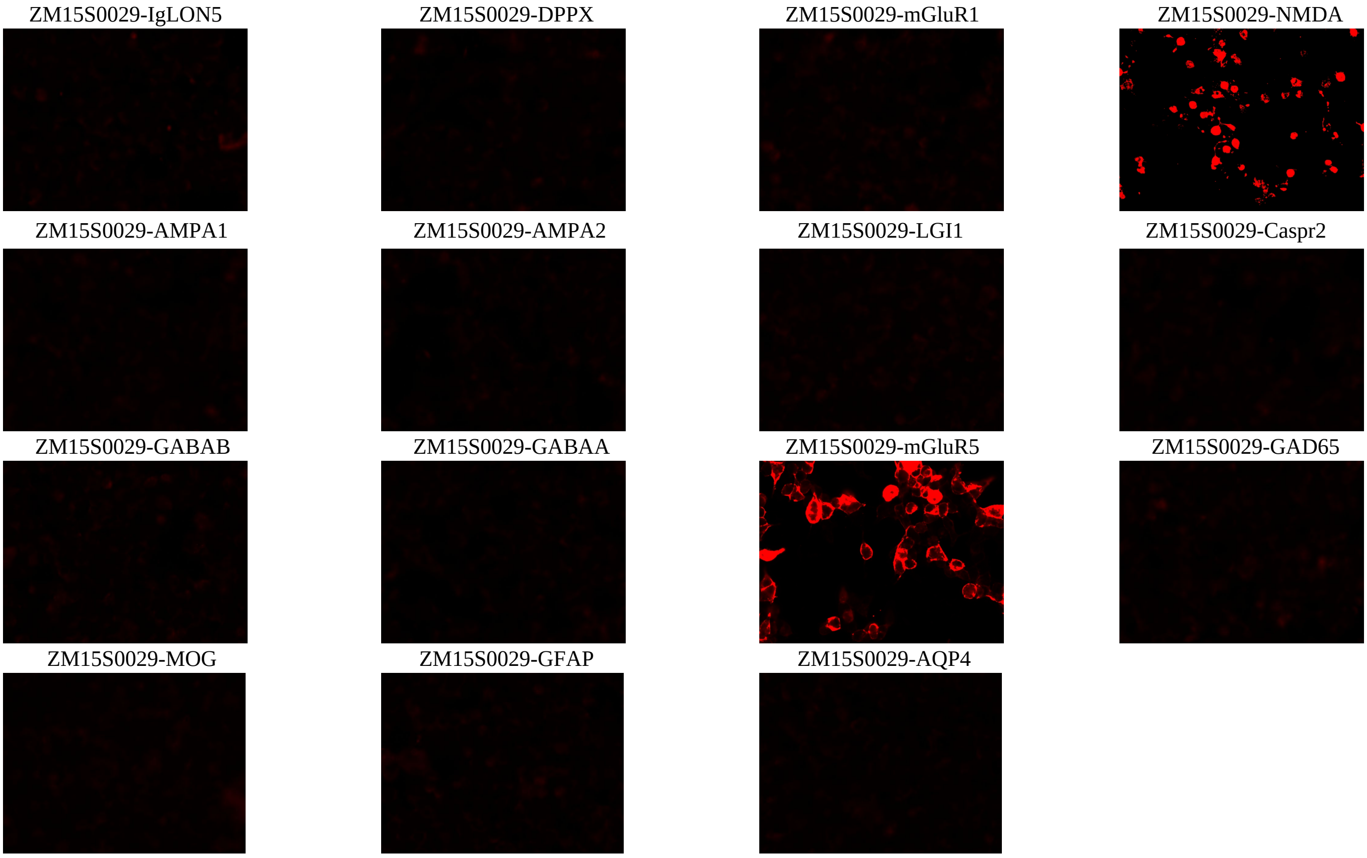

Figure B： The results of the fluorescence map of the sample.

Test Results: The results of the red channel image are displayed, and the negative or positive results are first determined according to the above judgment criteria. If the sample is a positive result, 3-5 fields are selected under the microscope and compared with the red fluorescence of the control sample to give the positive titer value by comparing the intensity of the red fluorescence with that of the control sample.

CBAIIFT Result interpretation (serum)		
Antibody titers results	The 1:10 dilution ratio can be observed to have a fluorescence intensity.	Compare the fluorescence intensity with the positive standard of 1:100 and 1:320 titer.
negative	Negative	-------
1:10	Pianissimo	Fluorescence intensity far＜1:100
1:30	Piano	Fluorescence intensity＜1:100
1:100	Strong	Fluorescence intensity=1:100
1:300	Fortissimo	Fluorescence intensity=1:300
1:1000	Fortissimo	Fluorescence intensity＞1:300

CBAIIFT Result interpretation (CSF)		
Antibody titers results	The un-diluted condition can be observed in the fluorescence intensity.	Compare the fluorescence intensity with the positive standard of 1:100 and 1:320 titer.
negative	Negative	-------
1:1	Pianissimo	Fluorescence intensity far＜1:100
1:10	Piano	Fluorescence intensity＜1:100
1:30	Moderate	Fluorescence intensity＜1:100
1:100	Strong	Fluorescence intensity=1:100
1:300	Fortissimo	Fluorescence intensity=1:300
1:1000	Fortissimo	Fluorescence intensity＞1:300

Main Check 侯莉 Reviewer [illegible] Authorize [illegible] Primary Testing Laboratory: Minte Biology

Test report Date: June 18, 2022

Website: www.kingmed.com.cn

GZ002MNSU3KYEDJ
